# Abemaciclib in Combination With Endocrine Therapy for Patients With Hormone Receptor-Positive, HER2-Negative Metastatic Breast Cancer: A Phase 1b Study

**DOI:** 10.3389/fonc.2021.810023

**Published:** 2022-02-10

**Authors:** Sara M. Tolaney, Muralidhar Beeram, J. Thaddeus Beck, Alison Conlin, E. Claire Dees, Shannon L. Puhalla, Brent N. Rexer, Howard A. Burris, Komal Jhaveri, Teresa Helsten, Carlos Becerra, Kevin Kalinsky, Kathleen N. Moore, Allison M. Manuel, Andrew Lithio, Gregory L. Price, Sonya C. Chapman, Lacey M. Litchfield, Matthew P. Goetz

**Affiliations:** ^1^ Department of Medical Oncology, Dana-Farber Cancer Institute, Boston, MA, United States; ^2^ South Texas Accelerated Research Therapeutics, San Antonio, TX, United States; ^3^ Department of Medical Oncology and Hematology, Highlands Oncology, Springdale, AR, United States; ^4^ Providence Cancer Center, Portland, OR, United States; ^5^ Lineberger Comprehensive Cancer Center, University of North Carolina at Chapel Hill, Chapel Hill, NC, United States; ^6^ UPMC Hillman Cancer Center, University of Pittsburgh, Pittsburgh, PA, United States; ^7^ Department of Medicine, Vanderbilt University Medical Center, Nashville, TN, United States; ^8^ Sarah Cannon Research Institute/Tennessee Oncology, Nashville, TN, United States; ^9^ Department of Medicine, Memorial Sloan Kettering Cancer Center, New York, NY, United States; ^10^ Department of Medicine, Weil Cornell Medical College, New York, NY, United States; ^11^ Moores Cancer Center, University of California San Diego, San Diego, CA, United States; ^12^ Texas Oncology, Dallas, TX, United States; ^13^ Department of Medicine, Columbia University, New York, NY, United States; ^14^ Department of Hematology/Oncology, Emory University Winship Cancer Institute, Atlanta, GA, United States; ^15^ Stevenson Cancer Center, University of Oklahoma Health Sciences Center, Oklahoma City, OK, United States; ^16^ Sarah Cannon Research Institute, Nashville, TN, United States; ^17^ Eli Lilly and Company, Indianapolis, IN, United States; ^18^ Department of Oncology, Mayo Clinic, Rochester, MN, United States; ^19^ Department of Molecular Pharmacology and Experimental Therapeutics, Mayo Clinic, Rochester, MN, United States

**Keywords:** abemaciclib, metastatic breast cancer, CDK4, CDK6, endocrine therapy

## Abstract

**Background:**

Cyclin-dependent kinases (CDK) 4 and 6 regulate G1 to S cell cycle progression and are often altered in cancers. Abemaciclib is a selective inhibitor of CDK4 and CDK6 approved for administration on a continuous dosing schedule as monotherapy or as combination therapy with an aromatase inhibitor or fulvestrant in patients with advanced or metastatic breast cancer. This Phase 1b study evaluated the safety and tolerability, pharmacokinetics, and antitumor activity of abemaciclib in combination with endocrine therapy for metastatic breast cancer (MBC), including aromatase inhibitors (letrozole, anastrozole, or exemestane) or tamoxifen.

**Patients and Methods:**

Women ≥18 years old with hormone receptor positive (HR+), human epidermal growth factor receptor 2 negative (HER2-) MBC were eligible for enrollment. Eligibility included measurable disease or non-measurable but evaluable bone disease by Response Evaluation Criteria in Solid Tumours (RECIST) v1.1, Eastern Cooperative Oncology Group performance status 0–1, and no prior chemotherapy for metastatic disease. Adverse events were graded by the National Cancer Institute Common Terminology Criteria for Adverse Events v4.0 and tumor response were assessed by RECIST v1.1.

**Results:**

Sixty-seven patients were enrolled and received abemaciclib 200 mg every 12 hours in combination with letrozole (Part A, n=20), anastrozole (Part B, n=16), tamoxifen (Part C, n=16), or exemestane (Part D, n=15). The most common treatment-emergent adverse events (TEAE) were diarrhea, fatigue, nausea, and abdominal pain. Grade 4 TEAEs were reported in five patients (one each with hyperglycemia, hypertension, neutropenia, procedural hemorrhage, and sepsis). There was no effect of abemaciclib or endocrine therapy on the pharmacokinetics of any combination study drug. Across all treated patients, the median progression-free survival was 25.4 months (95% confidence interval: 18.0, 35.8). The objective response rate was 38.9% in 36 patients with measurable disease.

**Conclusions:**

Abemaciclib in combination with multiple endocrine therapy options exhibited manageable safety and promising antitumor activity in patients with HR+, HER2- MBC.

**Clinical Trial Registration:**

https://clinicaltrials.gov/, identifier NCT02057133

## Introduction

Endocrine therapy (ET) forms the foundation of treatment for hormone receptor positive (HR+), human epidermal growth factor receptor 2 negative (HER2-) advanced breast cancer. Resistance to this therapy often occurs, however, resulting in the need to identify strategies to improve clinical outcomes. Many HR+ breast cancers demonstrate overexpression of cyclin D1, which interacts directly with cyclin-dependent kinase (CDK) 4 and 6 in an active protein complex that promotes cell proliferation (1, 2). CDK4 and CDK6, therefore, emerged as viable therapeutic targets, and evaluation of CDK4 and 6 inhibitors in combination with other therapies is an area of active investigation that is changing the treatment landscape for HR+, HER2- advanced breast cancer.

Abemaciclib is an oral, potent, and selective small-molecule inhibitor of CDK4 and CDK6 with greater potency against CDK4 than CDK6 (3). Abemaciclib exhibited activity as monotherapy or in combination with ET or chemotherapy in preclinical studies (3–5). A Phase 1 study (NCT01394016) initially demonstrated early evidence of the clinical activity and tolerability of abemaciclib in a heavily pretreated population with refractory HR+, HER2- advanced breast cancer, either as monotherapy or in combination with fulvestrant (6). Subsequent Phase 3 studies evaluated abemaciclib in combination with nonsteroidal aromatase inhibitors (NSAI; letrozole or anastrozole) as initial therapy (MONARCH 3), or with fulvestrant after progression on ET (MONARCH 2), leading to approval of abemaciclib in combination with these ETs for the treatment of advanced or metastatic breast cancer (7–9). A Phase 2 study evaluating abemaciclib as monotherapy following ET and prior chemotherapy (MONARCH 1) also led to approval for that indication (10).

In addition to these studies, the multicenter, open-label Phase 1b study presented here evaluated the safety and tolerability, pharmacokinetics, and antitumor activity of abemaciclib when administered orally in combination with multiple ET options, including NSAI (letrozole, anastrozole) as well as a steroidal aromatase inhibitor (exemestane) or tamoxifen in patients with HR+, HER2 metastatic breast cancer (MBC).

## Materials and Methods

### Study Design and Objectives

This was a Phase 1b, multicenter, nonrandomized, open-label study of abemaciclib in combination with other therapies for patients with either HR+, HER2- MBC (Parts A, B, C, D, E, G, and I) or HER2+ MBC (Parts F and H). Here we report only on Parts A–D; the remaining cohorts will be reported separately. Patients were enrolled at 14 sites in the United States from March 18, 2014 to January 14, 2015.

The primary objective was to evaluate the safety and tolerability of abemaciclib when administered orally in combination with letrozole, anastrozole, tamoxifen, or exemestane in patients with HR+, HER2- MBC. The secondary objectives included assessment of the pharmacokinetics, antitumor activity, and changes in patient-reported symptom burden with abemaciclib and corresponding ET when given in combination.

### Patients

**Figure 1:**
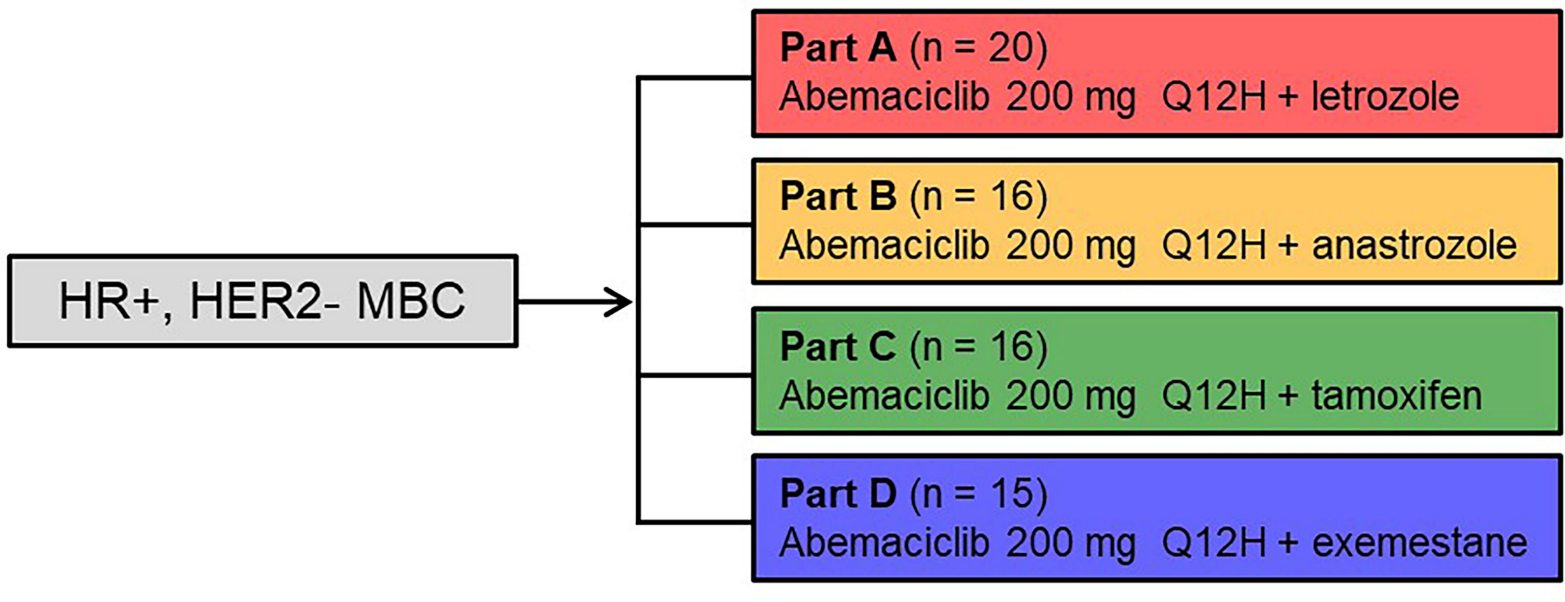
Study Design: Parts A–D. Women ≥18 years of age diagnosed with hormone receptor positive, human epidermal growth factor receptor 2 negative metastatic breast cancer received abemaciclib at 200 mg orally every 12 hours and the assigned combination endocrine therapy (letrozole 2.5 mg – Part A, anastrozole 1 mg – Part B, tamoxifen 20 mg – Part C, or exemestane 25 mg – Part D once daily on days 1 to 28 of a 28-day cycle. HER2-, human epidermal growth factor receptor 2; HR+, hormone receptor positive; MBC, metastatic breast cancer; Q12H, every 12 hours.

**Figure 1** annotates the study schema for the treatment regimens reported. Women ≥18 years of age diagnosed with HR+, HER2- MBC were eligible for enrollment in the study. Patients must have had either measurable disease or non-measurable but evaluable bone disease as defined by the Response Evaluation Criteria in Solid Tumours (RECIST) version 1.1. Additional inclusion criteria included: adequate organ function, an Eastern Cooperative Oncology Group performance status of ≤1, and an estimated life expectancy ≥12 weeks. Patients must have discontinued all previous therapies for breast cancer, except for ongoing corresponding combination therapy (Part A-letrozole; Part B-anastrozole; Part C-tamoxifen; and Part D-exemestane), and recovered from the acute effects of therapy. Prior systemic ET for metastatic disease was allowed for Part C-tamoxifen and required for Part D-exemestane (≥1 NSAI [anastrozole, letrozole] for metastatic disease); while prior systemic therapy for metastatic disease was not allowed for Parts A and B (except for ongoing letrozole in Part A or anastrozole in Part B). [Of note, prior neoadjuvant and/or adjuvant therapy was allowed.] Patients must have either been postmenopausal or premenopausal with ovarian suppression using a luteinizing hormone-releasing hormone agonist. Patients were excluded from the study if they were diagnosed with MBC with visceral crisis, lymphangitic spread, or leptomeningeal carcinomatosis; had brain metastasis without prior radiotherapy; received prior systemic chemotherapy for metastatic disease (patients may have received chemotherapy in the neoadjuvant or adjuvant setting) or prior therapy with a CDK4 and CDK6 inhibitor; or had been intolerant to the standard therapy drugs administered in the specific part of the study.

### Treatment and Dosing

Patients enrolled into Parts A–D received abemaciclib 200 mg orally every 12 hours (Q12H) and the assigned combination ET (Part A-letrozole 2.5 mg, Part B-anastrozole 1 mg, Part C-tamoxifen 20 mg, or Part D-exemestane 25 mg) once daily on days 1 to 28 of a 28-day cycle.

### Pharmacokinetics

Plasma samples were collected on Cycle 1 Day 1 and Cycle 2 Day 1 at 1, 2, 4, 6, 8, and 10 hours after study drug administration. In addition, pre-dose samples were collected on Cycle 1 Day 1, Cycle 1 Day 15, and Cycle 2 Day 1. Samples were analyzed for abemaciclib (and its metabolites), letrozole, anastrozole, tamoxifen, and exemestane using liquid chromatography with tandem mass spectrometry method. Standard noncompartmental analysis using Phoenix WinNonlin 6.3 (Pharsight Corporation) was used to assess patients who received at least one dose of the study drug. The primary parameters for analysis include maximum concentration (Cmax) and area under the concentration-time curve (AUC0-tlast, AUC0-tau) using the log linear trapezoidal method.

### Safety Assessments

Standard laboratory tests (e.g., chemistry and hematology panels) and serum pregnancy tests for females with child-bearing potential were performed. Adverse events (AE) were graded for severity using the National Cancer Institute Common Terminology Criteria for Adverse Events Version 4.0.

### Patient-Reported Outcomes

Patient-reported data were collected at baseline, each on-study visit, and the follow-up visit using the 19-item (13 symptoms plus six interferences) MD Anderson Symptom Inventory (MDASI) instrument. MDASI uses a numeric rating scale, and response options range from 0 (not present or did not interfere) to 10 (as bad as you can imagine/interfered completely). Mean change from baseline scores for symptom and interference items (post-baseline visits) were evaluated.

### Statistical Analysis

The analysis of this study was descriptive, and no hypothesis was tested. Data analyses were provided by study part and dose group. Summary statistics for continuous variables included number of patients, mean, median, standard deviation, minimum, and maximum. Categorical endpoints were summarized using number of patients, frequency, and percentages. Change in lesion data from baseline in the sum of target lesion size was listed by cycle and depicted as a waterfall plot. Overall response is derived based on investigator-assessed response using RECIST version 1.1 and summarized in terms of: objective response rate [ORR; complete response (CR) + partial response (PR)], disease control rate [DCR; CR + PR + stable disease (SD)], and clinical benefit rate (CBR; CR + PR + SD ≥24 weeks). Progression-free survival (PFS) and duration of response (DoR) were estimated by Kaplan-Meier method.

### Ethics Approval Statement

The study protocol was reviewed and approved by the institutional review board at participating sites and was conducted in accordance with international ethics guidelines. This included the Declaration of Helsinki and Council for International Organizations of Medical Sciences International Ethical Guidelines, International Conference on Harmonisation Good Clinical Practices Guidelines, and other applicable laws and regulations. All patients signed the approved consent forms for the study.

## Results

### Patients

A total of 67 patients with HR+, HER2- MBC were enrolled in Parts A-D of the study: Part A-letrozole (n=20); Part B-anastrozole (n=16); Part C-tamoxifen (n=16); and Part D-exemestane (n=15) (**Figure 1**). Patients received treatment from June 26, 2014 with a data cut-off of April 2, 2018. At the time of data cut-off, 11 patients continued to receive study treatment. Baseline patient and disease characteristics are summarized in **Table 1**. Most patients were White (64, 95.5%) and aged <65 years (51, 76.1%), with a median age of 57 (range: 28–77). Thirty-six patients (53.7%) had measurable disease at study entry (RECIST v1.1). The median prior systemic therapies across multiple settings were three (range: 1–8). Eleven patients in Parts A, 5 patients in Part B, 7 patients in Part C, and 1 patient in Part D received letrozole, anastrozole, tamoxifen, or exemestane, respectively, in the metastatic setting prior to study enrolment (includes those who started the corresponding drug at least 30 days prior to receiving study drug).

**Table 1:** Baseline Patient and Disease Characteristics.

	Letrozole Part A (N=20)	Anastrozole Part B (N=16)	Tamoxifen Part C (N=16)	Exemestane Part D (N=15)	Parts A–D(N=67)
Age in years, median (range)	59.0 (33–73)	55.5 (28–72)	59.5 (46–77)	52.0 (40–73)	57.0 (28–77)
≥65, n (%)	6 (30.0)	3 (18.8)	3 (18.8)	4 (26.7)	16 (23.9)
Race, n (%)					
White	20 (100.0)	14 (87.5)	16 (100.0)	14 (93.3)	64 (95.5)
All other	0 (0.0)	2 (12.5)	0 (0.0)	1 (6.7)	3 (4.5)
Eastern Cooperative Oncology Group performance status, n (%)					
0	16 (80.0)	13 (81.3)	12 (75.0)	12 (80.0)	53 (79.1)
1	3 (15.0)	3 (18.8)	4 (25.0)	3 (20.0)	13 (19.4)
2	1 (5.0)	0 (0.0)	0 (0.0)	0 (0.0)	1 (1.5)
Visceral disease n (%) (lung, liver and/or brain)	5 (25.0)	6 (37.5)	5 (31.3)	6 (40.0)	22 (32.8)
Number of metastatic sites, n (%)					
1	6 (30.0)	4 (25.0)	3 (18.8)	2 (13.3)	15 (22.4)
2	8 (40.0)	3 (18.8)	3 (18.8)	5 (33.3)	19 (28.4)
≥3	6 (30.0)	9 (56.3)	10 (62.5)	8 (53.3)	33 (49.3)
Prior systemic therapies, median (range)^a^	2 (1–4)	3 (1–8)	3 (1–8)	4 (1–6)	3 (1–8)
Measurable disease, n (%)	9 (45.0)	9 (56.3)	8 (50.0)	10 (66.7)	36 (53.7)

a Includes chemotherapy, endocrine therapy, adjuvant therapies, neoadjuvant therapies, and metastatic therapies.

### Safety

All patients had at least one treatment-emergent AE (TEAE). The most common TEAEs of any grade, regardless of causality, were diarrhea (98.5%), fatigue (83.6%), nausea (74.6%), abdominal pain (50.7%), vomiting (44.8%), decreased appetite (40.3%), neutropenia (35.8%), alopecia (31.3%), and anemia (31.3%; **Table 2**). Across all parts, grade 3 TEAEs occurred in 44 patients (65.7%). The most common grade 3 TEAEs were diarrhea (35.8%), fatigue (20.9%), and neutropenia (20.9%). Grade 2/3 diarrhea most commonly occurred during the first two cycles of treatment. Grade 4 TEAEs occurred in five patients (2.9%; one each with hyperglycemia, hypertension, neutropenia, procedural hemorrhage, and sepsis). Of these, only neutropenia was considered possibly related to study treatment. No patients experienced a grade 5 TEAE.

**Table 2:** Treatment-emergent Adverse Events, Regardless of Causality.

Adverse Event^a^	Letrozole Part A (N=20)		Anastrozole Part B (N=16)		Tamoxifen Part C (N=16)		Exemestane Part D (N=15)		Parts A–D(N=67)	
	Any Graden (%)	Grade ≥3n (%)	Any Graden (%)	Grade ≥3n (%)	Any Graden (%)	Grade ≥3n (%)	Any Graden (%)	Grade ≥3n (%)	Any Graden (%)	Grade ≥3n (%)
Any adverse event	20 (100.0)	14 (70.0)	16 (100.0)	15 (93.8)	16 (100.0)	12 (75.0)	15 (100.0)	8 (53.3)	67 (100.0)	49 (73.1)
Diarrhea	20 (100.0)	10 (50.0)	16 (100.0)	5 (31.3)	16 (100.0)	5 (31.3)	14 (93.3)	4 (26.7)	66 (98.5)	24 (35.8)
Fatigue	18 (90.0)	4 (20.0)	13 (81.3)	3 (18.8)	13 (81.3)	5 (31.3)	12 (80.0)	2 (13.3)	56 (83.6)	14 (20.9)
Nausea	15 (75.0)	3 (15.0)	14 (87.5)	0 (0.0)	11 (68.8)	1 (6.3)	10 (66.7)	0 (0.0)	50 (74.6)	4 (6.0)
Abdominal pain	7 (35.0)	1 (5.0)	8 (50.0)	0 (0.0)	10 (62.5)	0 (0.0)	9 (60.0)	3 (20.0)	34 (50.7)	4 (6.0)
Vomiting	9 (45.0)	2 (10.0)	7 (43.8)	1 (6.3)	7 (43.8)	0 (0.0)	7 (46.7)	0 (0.0)	30 (44.8)	3 (4.5)
Decreased appetite	10 (50.0)	1 (5.0)	8 (50.0)	0 (0.0)	5 (31.3)	0 (0.0)	4 (26.7)	0 (0.0)	27 (40.3)	1 (1.5)
Neutropenia	9 (45.0)	4 (20.0)	9 (56.3)	6 (37.5)	2 (12.5)	2 (12.5)	4 (26.7)	3 (20.0)	24 (35.8)	15 (22.4)
Alopecia	5 (25.0)	0 (0.0)	6 (37.5)	0 (0.0)	5 (31.3)	0 (0.0)	5 (33.3)	0 (0.0)	21 (31.3)	0 (0.0)
Anemia	5 (25.0)	0 (0.0)	5 (31.3)	0 (0.0)	8 (50.0)	1 (6.3)	3 (20.0)	0 (0.0)	21 (31.3)	1 (1.5)
Arthralgia	3 (15.0)	0 (0.0)	8 (50.0)	1 (6.3)	4 (25.0)	0 (0.0)	5 (33.3)	0 (0.0)	20 (29.9)	1 (1.5)
Cough	5 (25.0)	0 (0.0)	5 (31.3)	0 (0.0)	4 (25.0)	0 (0.0)	6 (40.0)	0 (0.0)	20 (29.9)	0 (0.0)
Dehydration	3 (15.0)	0 (0.0)	6 (37.5)	1 (6.3)	4 (25.0)	2 (12.5)	3 (20.0)	1 (6.7)	16 (23.9)	4 (6.0)
Headache	2 (10.0)	0 (0.0)	4 (25.0)	1 (6.3)	4 (25.0)	0 (0.0)	5 (33.3)	0 (0.0)	15 (22.4)	1 (1.5)
Dyspnea	2 (10.0)	0 (0.0)	4 (25.0)	0 (0.0)	3 (18.8)	0 (0.0)	5 (33.3)	0 (0.0)	14 (20.9)	0 (0.0)
Flatulence	2 (10.0)	0 (0.0)	3 (18.8)	0 (0.0)	3 (18.8)	0 (0.0)	6 (40.0)	0 (0.0)	14 (20.9)	0 (0.0)
										

a Treatment-emergent adverse events that occurred in >20% of patients in at least one part of the study are listed. There were no grade 4 or 5 events reported in these categories except grade 4 neutropenia reported for one patient in Part B.

Eleven (16.4%) patients discontinued treatment due to AEs (**Table 3**), with diarrhea as the most commonly reported AE leading to treatment discontinuation (five patients, 7.5%). The most commonly reported SAEs, regardless of causality, were dehydration (four patients total, 6.0%; Parts B-anastrozole, C-tamoxifen, and D-exemestane), diarrhea (two patients, 3.0%; Part A-letrozole), and skin infection (two patients, 3.0%; Part B-anastrozole). Serious adverse events considered possibly related to study treatment occurred in seven patients (10.4%), and included dehydration (three patients total, 4.5%; Parts C-tamoxifen and D-exemestane), diarrhea (two patients, 3.0%; Part A-letrozole), and one patient each (1.5%) with neutropenia (Part B-anastrozole), obliterative bronchiolitis (Part B-anastrozole), and stomatitis (Part A-letrozole). No patient deaths due to AEs were reported up to the data cutoff date.

**Table 3:** Patient Disposition.

n (%)	Letrozole Part A (N=20)	Anastrozole Part B (N=16)	Tamoxifen Part C (N=16)	Exemestane Part D (N=15)	Parts A–D N=67
On treatment	1 (5.0)	5 (31.8)	3 (18.8)	2 (13.3)	11 (16.4)
Discontinued treatment	19 (95.0)	11 (68.8)	13 (81.3)	13 (86.7)	56 (83.6)
Reason for treatment discontinuation					
Adverse event^a^	6 (30.0)	3 (18.8)	1 (6.3)	1 (6.7)	11 (16.4)
Diarrhea	4 (20.0)	0 (0.0)	0 (0.0)	1 (6.7)	5 (7.5)
Death	0 (0.0)	0 (0.0)	0 (0.0)	0 (0.0)	0 (0.0)
Non-compliance with study drug	0 (0.0)	0 (0.0)	0 (0.0)	1 (6.7)	1 (1.5)
Physician decision	2 (10.0)	0 (0.0)	2 (12.5)	1 (6.7)	5 (7.5)
Progressive disease	8 (40.0)	7 (43.8)	8 (50.0)	8 (53.3)	31 (46.3)
Withdrawal by subject	3 (15.0)	1 (6.3)	2 (12.5)	2 (13.3)	8 (11.9)

a Adverse events of ≥10% incidence in at least one part of the study are listed.

### Dose Adjustments

More than half of patients (68.7%) had at least 1 dose reduction (**Table 4**). Part B-anastrozole had the greatest incidence (87.5%) of dose reduction, and Part D-exemestane had the least (53.3%). Across Parts A–D, 31.3% of patients had one dose reduction (to 150 mg Q12H), 29.9% had two (to 100 mg Q12H), and 7.5% had three (to 50 mg Q12H). All dose reductions occurred as a result of AEs, most commonly diarrhea (55.3%), fatigue (17.1%), or neutropenia (5.3%).

**Table 4:** Dose Adjustments and Omissions for Abemaciclib.

n (%)	Letrozole Part A (N=20)	Anastrozole Part B (N=16)	Tamoxifen Part C (N=16)	Exemestane Part D (N=15)	Parts A–D(N=67)
Patients with ≥1 dose adjustment or omission	16 (80.0)	16 (100.0)	14 (87.5)	11 (73.3)	57 (85.1)
Patients with dose reduction	12 (60.0)	14 (87.5)	12 (75.0)	8 (53.3)	46 (68.7)
1 dose reduction	4 (20.0)	5 (31.3)	8 (50.0)	4 (26.7)	21 (31.3)
2 dose reductions	7 (35.0)	7 (43.8)	3 (18.8)	3 (20.0)	20 (29.9)
≥3 dose reductions	1 (5.0)	2 (12.5)	1 (6.3)	1 (6.7)	5 (7.5)
Total dose reductions	21	25	17	13	76
Reason for dose reduction					
Adverse event^a^	21 (100.0)	25 (100.0)	17 (100.0)	13 (100.0)	76 (100.0)
Diarrhea	15 (71.4)	11 (44.0)	8 (47.1)	8 (61.5)	42 (55.3)
Fatigue	5 (23.8)	3 (12.0)	4 (23.5)	1 (7.7)	13 (17.1)
Neutropenia	NR	3 (12.0)	NR	1 (7.7)	4 (5.3)
Patients with dose omission	16 (80.0)	16 (100.0)	14 (87.5)	11 (73.3)	57 (85.1)
Total dose omissions	130	127	95	96	448
Dose omission duration (days); median (range)	1.0 (1–14)	1.0 (1–29)	1.0 (1–20)	1.0 (1–21)	1.0 (1–29)
Reason for dose omission					
Adverse event^a^	50 (38.5)	50 (39.4)	44 (46.3)	42 (43.8)	186 (41.5)
Diarrhea	24 (18.5)	12 (9.4)	19 (20.0)	28 (29.2)	83 (18.5)
Other^b^	77 (59.2)	77 (60.6)	50 (52.6)	52 (54.2)	256 (57.1)
Missing	3 (2.3)	0	1 (1.1)	2 (2.1)	6 (1.3)

a Adverse events of ≥10% incidence in at least one part of the study are listed.

b Other includes scheduling conflict or treatment availability.

NR, not reported.

Dose omissions were reported for most patients (85.1%; **Table 4**) and were typically brief, lasting only one day (median; **Table 4**). The most common reasons for dose omissions were scheduling conflicts (55.8%) and AEs (41.5%; most commonly diarrhea [18.5%]).

### Efficacy

For the 67 patients in Parts A–D (including measurable or non-measurable disease), one CR (Part D-exemestane) and 13 PR were observed, for a confirmed ORR of 20.9% (**Table 5**). The ORR was 10.0% in Part A-letrozole (two of 20 patients), 18.8% in Part B-anastrozole (three of 16), 18.8% in Part C-tamoxifen (three of 16) and 40.0% in Part D-exemestane (six of 15). DCR was 60% in Part A-letrozole (12 of 20 patients), 87.5% in Part B-anastrozole (14 of 16), 75.0% in Part C-tamoxifen (12 of 16), and 73.3% in Part D-exemestane (11 of 15). CBR was 40% in Part A-letrozole (8 of 20 patients), 81.3% in Part B-anastrozole (13 of 16), 75.0% in Part C-tamoxifen (12 of 16), and 60.0% in Part D-exemestane (9 of 15).

**Table 5:** Best Overall Response in Patients with Measurable and Non-measurable Disease by Response Evaluation Criteria in Solid Tumours v1.1.

Best Overall Response^a^	Letrozole Part A, n (%)	Anastrozole Part B, n (%)	Tamoxifen Part C, n (%)	Exemestane Part D, n (%)	Parts A–Dn (%)
All patients (N)	20	16	16	15	67
CR	0 (0.0)	0 (0.0)	0 (0.0)	1 (6.7)	1 (1.5)
PR	2 (10.0)	3 (18.8)	3 (18.8)	5 (33.3)	13 (19.4)
SD	10 (50.0)	11 (68.8)	9 (56.3)	5 (33.3)	35 (52.2)
Progressive disease	2 (10.0)	1 (6.3)	3 (18.8)	2 (13.3)	8 (11.9)
Not assessed	6 (30.0)	1 (6.3)	1 (6.3)	2 (13.3)	10 (14.9)
Objective response rate (CR + PR)	2 (10.0)	3 (18.8)	3 (18.8)	6 (40.0)	14 (20.9)
Disease control rate (CR + PR + SD)	12 (60.0)	14 (87.5)	12 (75.0)	11 (73.3)	49 (73.1)
Clinical benefit rate (CR + PR + SD≥24 weeks)	8 (40.0)	13 (81.3)	12 (75.0)	9 (60.0)	42 (62.7)
Measurable disease (N)	9	9	8	10	36
CR	0 (0.0)	0 (0.0)	0 (0.0)	1 (10.0)	1 (2.8)
PR	2 (22.2)	3 (33.3)	3 (37.5)	5 (50.0)	13 (36.1)
SD	4 (44.4)	4 (44.4)	2 (25.0)	1 (10.0)	11 (30.6)
Progressive disease	2 (22.2)	1 (11.1)	3 (37.5)	1 (10.0)	7 (19.4)
Not assessed	1 (11.1)	1 (11.1)	0 (0.0)	2 (20.0)	4 (11.1)
Objective response rate (CR + PR)	2 (22.2)	3 (33.3)	3 (37.5)	6 (60.0)	14 (38.9)
Disease control rate (CR + PR + SD)	6 (66.7)	7 (77.8)	5 (62.5)	7 (70.0)	25 (69.4)
Clinical benefit rate (CR + PR + SD≥24 weeks)	3 (33.3)	6 (66.7)	5 (62.5)	6 (60.0)	20 (55.6)
PFS [at 6 months, % (95% CI)]	76.2 (42.7, 91.7)	86.7 (56.4, 96.5)	73.3 (43.6, 89.1)	75.2 (40.7, 91.4)	77.9 (64.3, 86.8)
Median PFS, months (95% CI)	28.5 (2.1, NE)	32.0 (9.7, NE)	18.4 (2.1, NE)	34.3 (5.6, NE)	25.4 (18.0, 35.8)

CI, confidence interval; CR, complete response; N, number of patients in population; n, number of patients in the category; NE, not estimable; PFS, progression free survival; PR, partial response; SD, stable disease.

a Response according to Response Evaluation Criteria in Solid Tumours version 1.1.

**Figure 2:**
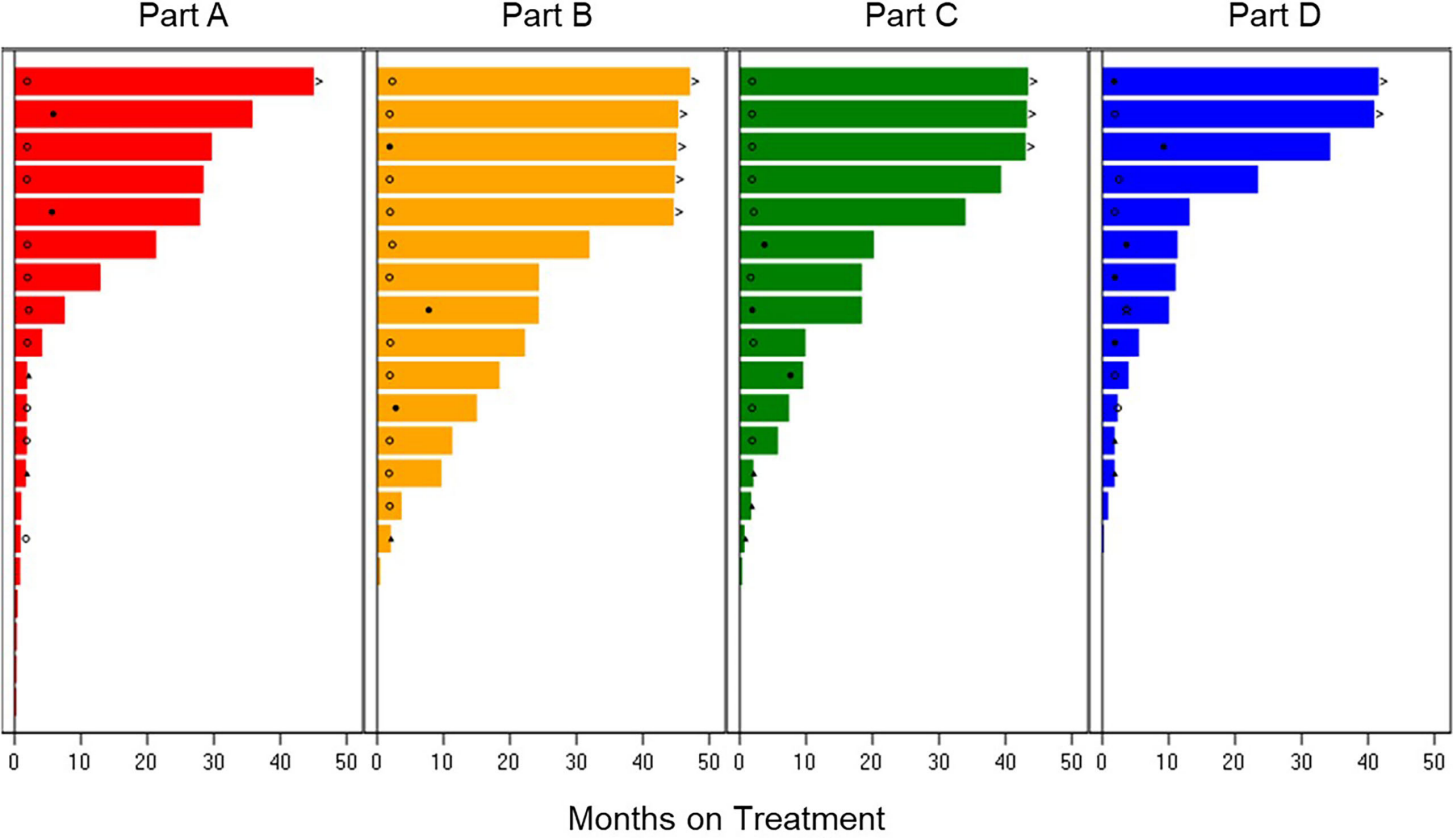
Treatment Duration and Best Overall Response. Time on treatment for patients receiving abemaciclib in combination with letrozole Part A, anastrozole Part B, tamoxifen Part C, or exemestane Part D. Best overall response is indicated as: star = complete response; filled circle = partial response; open circle = stable disease; filled triangle = progressive disease; diamond = not evaluable. The > sign indicates treatment ongoing at time of data cut-off.

**Figure 3:**
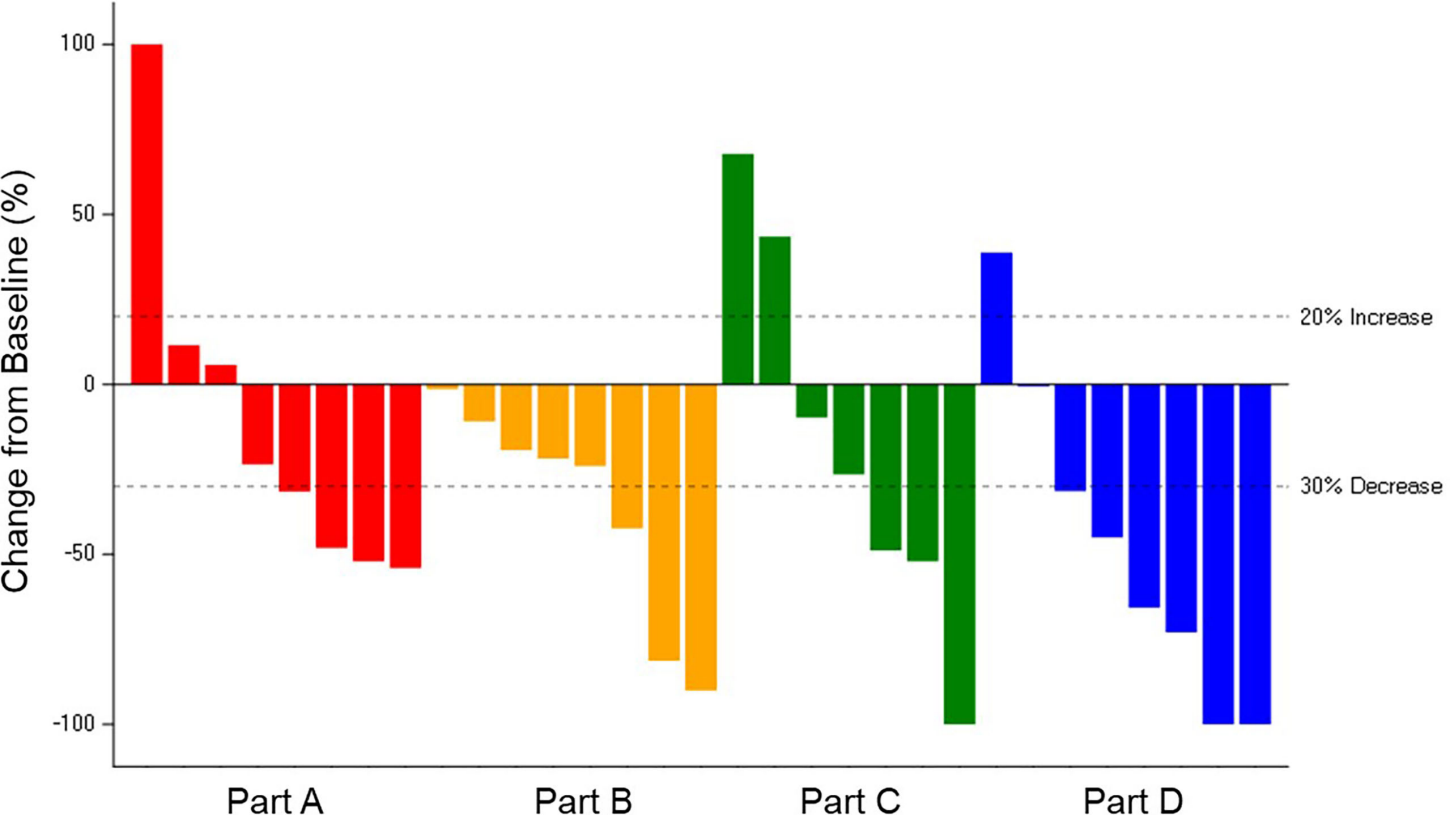
Change in Tumor Size for Patients with Measurable Disease. Best percent change in tumor size for 31 patients in Parts A–D with measurable disease and available post-baseline assessments, colored by response according to Response Evaluation Criteria in Solid Tumours v1.1. Change in tumor size greater than 100% is truncated at 100%. Comparison among study parts is not possible due to differences in patient and disease characteristics and because enrollment opened sequentially: Part A-letrozole and Part B- anastrozole → Part C-tamoxifen → Part D-exemestane.

The ORR was 38.9% for the 36 patients across Parts A–D with measurable disease: 22.2% in Part A-letrozole, 33.3% in Part B-anastrozole, 37.5% in Part C-tamoxifen, and 60.0% in Part D-exemestane. The median PFS was: 34.3 months in Part D-exemestane (95% confidence interval [CI]: 5.6, not estimable [NE]), 32.0 months in Part B-anastrozole (95% CI: 9.7, NE), 28.5 months in Part A-letrozole (95% CI: 2.1, NE), and 18.4 months in Part C-tamoxifen (95% CI: 2.1, NE); 11 patients remained on treatment at the time of data cut-off. Time on treatment and best overall responses are displayed in **Figure 2**. The median time to response in Parts A–D was 3.6 months, with a median DoR of 16.6 months (95% CI: 6.5, 30.1). The longest DoR was exhibited in Part B-anastrozole, with a maximum of more than 43.3 months and median DoR not reached. The 12-month DoR rate was 100.0% for Parts A-letrozole, B-anastrozole, and C-tamoxifen and 41.7% for Part D-exemestane. These results are displayed as a waterfall plot in **Figure 3**.

### Pharmacokinetics

**Figure 4:**
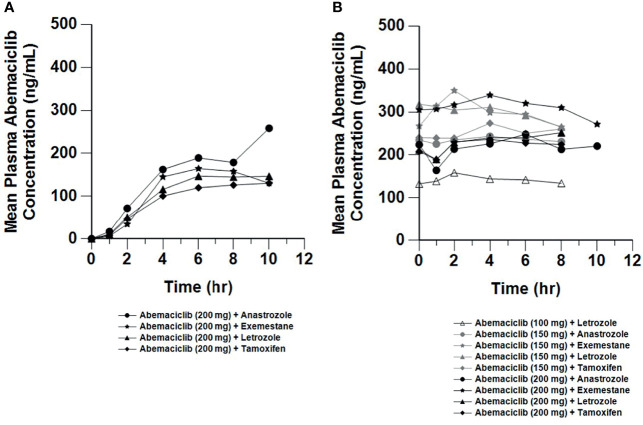
Mean Plasma Concentrations of Abemaciclib Following Single and Multiple Doses. Mean plasma concentration *vs.* time profiles of abemaciclib following oral administration of abemaciclib with combination therapies after a single abemaciclib dose (200 mg; **(A)** and on Cycle 2 Day 1 at steady state after multiple twice daily abemaciclib doses (100 to 200 mg; **(B)**. Plasma concentration data for abemaciclib and metabolites were available from 67 patients (n=20, letrozole; n=16, anastrozole; n=16, tamoxifen; n=15, exemestane). hr, hour.

Plasma concentration data were available from all patients who received the relevant study drug [abemaciclib and metabolites (n=67; **Figure 4**), letrozole (n=20; **Supplementary Figure 1A**), anastrozole (n=16; **Supplementary Figure 1B**), tamoxifen (n=16; **Supplementary Figure 1C**), exemestane (n=15; **Supplementary Figure 1D**)]. After a single 200 mg dose of abemaciclib, the mean Cmax ranged from 129 to 147 ng/mL and mean AUC(0-tlast) ranged from 608 to 770 (hr*ng/mL). Following 150 mg or 200 mg repeated doses, the steady-state, mean Cmax ranged from 185 to 332 ng/mL and AUC(0-tlast) ranged from 1280 to 2520 (hr*ng/mL).

### Patient-Reported Outcomes

Patient-reported MDASI items with the highest mean scores at baseline were fatigue, pain, disturbed sleep, feeling drowsy, interference with work, and interference with general activity. Overall for mean change from baseline, the greatest symptom worsening was observed in “lack of appetite” [0.70; 95% CI (0.18, 1.22)], and the greatest symptom improvement was observed for patient-reported “pain” [0.54; 95% CI (-1.06, -0.01)] (**Supplementary Figures 2A, B**). However, no clinically significant (1.2 points or greater) mean change from baseline was observed in the MDASI symptom or interference items (11).

## Discussion

Oral highly selective inhibitors of CDK4 and CDK6, such as abemaciclib, represent an important therapeutic advancement in HR+ breast cancer (12). Early evidence of a tolerable safety profile and antitumor activity was observed in a Phase 1 study, with abemaciclib administered either as a single agent or in combination with fulvestrant evaluating the 200 mg Q12H dose. This report explores the safety and antitumor activity of abemaciclib at the 200 mg Q12H dosing level in combination with multiple oral ETs, including letrozole, anastrozole, tamoxifen, or exemestane.

Diarrhea (98.5%, any grade) was the most commonly reported AE, which was consistent with other studies of abemaciclib, and grade 3 diarrhea was observed in 35.8% of patients using a starting dose of abemaciclib of 200 mg Q12H. The recommended dose of abemaciclib was reduced to 150 mg Q12H in subsequent Phase 3 trials combining abemaciclib with ET—in MONARCH 3, grade 3 diarrhea was observed in only 9.5% of patients receiving abemaciclib plus NSAI (7). In this Phase 1b study, grade 3 diarrhea was most common during the first two treatment cycles and diarrhea did not frequently lead to treatment discontinuation (five patients, 7.5%). Other common AEs included fatigue (83.6%), nausea (74.6%), and abdominal pain (50.7%). This is consistent with the safety profile observed in subsequent studies; besides diarrhea, the most common AEs of any grade reported in patients receiving abemaciclib plus NSAI in MONARCH 3 included neutropenia (41.3%), fatigue (40.1%), infections (39.1%), nausea (38.5%), and abdominal pain (29.1%) (7), although the rates of fatigue, nausea, and abdominal pain were higher in this study (Parts A-D). Similar to patients in Part C of our study, the most common AEs reported for patients receiving abemaciclib plus tamoxifen in the nextMONARCH study included diarrhea (53.8%), neutropenia (41.0%), anemia (39.7%), infections (32.1%), nausea (30.8%), fatigue (29.5%), leukopenia (26.9%), and abdominal pain (26.9%) (13). In Parts A-D of this Phase 1b study, no unexpected safety signals were observed, and treatment discontinuation due to AEs was not common (11 patients, 16.4%). Grade ≥3 AEs and SAEs considered possibly related to study treatment occurred in 42 patients (62.7%) and seven patients (10.4%), respectively, and there were no deaths due to AEs. Overall, a clinically significant change in patient-reported symptom and interference scores was not observed compared to the baseline MDASI assessment.

This study also evaluated the pharmacokinetics of abemaciclib, metabolites, and the combination agents letrozole, anastrozole, tamoxifen, and exemestane. The exposures of abemaciclib and metabolites were consistent across the combinations and were comparable to observations for abemaciclib as a single agent in the previous Phase 1 study (6). Similarly, the concentrations of the combination ET were also comparable to those observed in monotherapy studies (14, 15). This indicates that abemaciclib does not impact ET exposures, and equally that ET does not affect abemaciclib pharmacokinetics. This is consistent with the known clearance routes and lack of interaction potential between abemaciclib and anastrozole, letrozole, exemestane, and tamoxifen (14–16). At a dose of 150 mg Q12H, the mean steady state exposures of abemaciclib achieved in combination with anastrozole, letrozole, tamoxifen, or exemestane were consistent with the exposure associated with target inhibition in xenograft models (5).

When given in combination with ET (letrozole, anastrozole, tamoxifen, and exemestane) in this study, abemaciclib showed evidence of antitumor activity. For patients with measurable disease, the overall ORR was 38.9% (Parts A–D) and the overall CBR was 55.6%.

Subsequent studies have evaluated the efficacy and tolerability of abemaciclib in combination with ET in patients with advanced breast cancer or MBC, including fulvestrant (MONARCH 2), NSAI (MONARCH 3), and tamoxifen (nextMONARCH) (7, 8, 13). In the MONARCH 2 trial, abemaciclib plus fulvestrant significantly improved the PFS (median 16.4 *vs.* 9.3 months) and ORR (48.1% *vs.* 21.3%) compared with placebo plus fulvestrant and exhibited a good safety profile in patients with HR+, HER2– advanced breast cancer who had progressed while receiving ET (8). Treatment with abemaciclib plus fulvestrant also resulted in a median overall survival benefit of 9.4 months compared to placebo plus fulvestrant (17). In the MONARCH 3 study, abemaciclib plus a NSAI (letrozole or anastrozole) exhibited efficacy as initial therapy for HR+, HER2- advanced breast cancer, significantly improving PFS (median 28.2 *vs.* 14.8 months) and ORR (61.0% *vs.* 45.5%) compared to placebo plus NSAI, while maintaining a tolerable safety profile (7, 18). Abemaciclib plus tamoxifen was evaluated in a subsequent study in women with HR+, HER2- MBC who had received prior chemotherapy (nextMONARCH), with a median PFS of 9.1 months and an ORR and CBR of 34.6% and 61.5%, respectively (13). nextMONARCH was restricted to a more heavily pretreated population following ET and at least two chemotherapy regimens, whereas in Part C-tamoxifen of this study prior ET was allowed, but not required, and prior chemotherapy for metastatic disease was prohibited. The current study was the first to evaluate antitumor activity in a cohort of patients receiving abemaciclib in combination with exemestane.

The results from this study supported the favorable safety profile and efficacy of abemaciclib in combination with multiple ET options for patients with HR+, HER2- MBC. The promising efficacy outcomes from this small Phase 1b study were further supported across subsequent Phase 2 and 3 clinical trials.

## Data Availability Statement

The datasets presented in this article are not readily available because Eli Lilly and Company provides access to all individual participant data collected during the trial, after anonymization, except for pharmacokinetic or genetic data. Data are available to request 6 months after the indication studied has been approved in the USA and EU and after primary publication acceptance, whichever is later. No expiration date of data requests is currently set once data are made available. Access is provided after a proposal has been approved by an independent review committee identified for this purpose and after receipt of a signed data sharing agreement. Data and documents, including the study protocol, statistical analysis plan, clinical study report, and blank or annotated case report forms, will be provided in a secure data sharing environment. Requests to access the datasets should be directed to http://www.vivli.org.

## Ethics Statement

The studies involving human participants were reviewed and approved by Columbia University IRB, New York, NY Dana Farber Cancer Institute, The Office for Human Research Studies (OHRS), Boston, MA IntegReview IRB, Austin, TX Mayo Clinic Institutional Review Board, Rochester, MN Memorial Sloan Kettering Cancer Center, Institutional Review Board (IRB/PB), New York, NY Providence Health and Services IRB, Portland, OR University of California-San Diego, Human Research Protections Program, La Jolla, CA University of North Carolina at Chapel Hill, Office of Human Research Ethics, Chapel Hill, NC US Oncology Inc. IRB, The Woodlands, TX Vanderbilt University IRB, Nashville, TN Western Institutional Review Board (WIRB), Puyallup, WA. The patients/participants provided their written informed consent to participate in this study.

## Author Contributions

All authors contributed to the conception, design, or conduct of this study. All authors contributed to manuscript revision and read and approved the submitted version.

## Funding

This study was sponsored by Eli Lilly and Company.

## Conflict of Interest

Authors AMM, AL, GLP, SCC and LML were employed by Eli Lilly and Company. SMT received research grants and personal fees from AstraZeneca, Bristol-Myers Squibb, Cyclacel, Eisai, Eli Lilly (outside submitted work), Exelixis, Genentech/Roche, Gilead, Merck, Nektar, Nanostring, Odonate, Pfizer, Puma, Sanofi; and personal fees from 4D Pharma, Athenex, BeyondSpring Pharma, Certara, Chugai Pharma, CytomX, Daiichi-Sankyo, Ellipses Pharma, G1 Therapeutics, Kyowa Kirin Pharmaceuticals, Mersana Therapeutics, OncoPep, OncoSec, OncXerna, Infinity Therapeutics, Samsung Bioepsis Inc., Seattle Genetics, Zentalis, and Zymeworks. MB received honoraria or research funding paid to the institution from Agios, Eli Lilly, Genentech, Johnson & Johnson, Merck, Merrimack, Mersana, Puma Biotechnology, Phoenix Molecular Designs, Zymerworks; Speaker’s Bureau paid to the institution from Bristol-Meyer-Squib, Genentech, and Merck; and travel expenses from Genentech and Merck. Consulting or advisory role payment was paid to an immediate family member from Bayer, Merck, Novartis and Seattle Genetics. ECD acted in a consulting or advisory role for Novartis, Strata Oncology, G1 Therapeutics, received research funding from Novartis, Genentech/Roche, Pfizer, Merck, H3 Biomedicine, Meryx Pharmaceuticals, and received reimbursement for travel expenses from G1 Therapeutics. HAB received research grants paid to the institution from Abbvie, Agios, Arch Oncology, ARMO Biosciences, Array BioPharma, AstraZeneca, Bayer, BeiGene, BioAtla, BioMed Valley Discoveries, BioTheryX, Boehringer Ingelheim, Bristol-Myers Squibb, CALGB, CicloMed, Coordination Pharmaceuticals, CytomX, eFFECTOR Therapeutics, Foundation Medicine, Gossamer Bio, Lilly, EMD Serono, Roche/Genentech, Gilead Sciences, GlaxoSmithKline, Harpoon, Hengrui Therapeutics, Incyte, Janssen, Jounce, Kymab, MacroGenics, MedImmune, Merck, Millennium, Moderna, NGM Biopharmaceuticals, Novartis, Pfizer, Revolution Medicine, Ryvu Therapeutics, SeaGen, Tesaro, TG Therapeutics, Verastem, Vertex, XBiotech, Zymeworks; uncompensated consulting for AstraZeneca, Bayer, Boehringer Ingelheim, Bristol Myers Squibb, GRAIL, Incyte, Novartis, Vincerx Pharma; and is employed by HCA Healthcare/Sarah Cannon and has Stock ownership with HCA Healthcare/Sarah Cannon. MPG received research grants paid to the institution: Biovica, Context Pharm, Eli Lilly, Novartis, Pfizer Sermonix; grants to the institution from Eli Lilly and Pfizer; and personal feed from Genentech Health. KNM served on advisory boards for Astra Zeneca, Aravive, Alkemeres, Blueprint Pharma, Eisai/Serono, Genentech/Roche, GSK/Tesoro, Hengrui, Immunogen, Inxmed, IMab, Lilly, Mereo, Merck, Mersana, Myriad, Novartis, Onconova, OncXerna, Tarveda, vBL Therapeutics. I am on SC for Genentech/Roche, Immunogen, Mersana and VBL Therapeutics. KK acted in a consulting or advisory role for 4D Pharma, AstraZeneca, Cyclocel, Eisai, Eli-Lilly, Daiichi Sankyo, Immunomedics, Merck, Novartis, Oncosec, Pfizer, Puma, and Seattle Genetics; and spouse has stock for Array BioPharma, Grail, and Pfizer.

The remaining authors declare that the research was conducted in the absence of any commercial or financial relationships that could be construed as a potential conflict of interest.

The authors declare that this study received funding from Eli Lilly and Company. The funder had the following involvement with the study: at the time of the study AMM, AL, GLP, LML, and SCC were employees and shareholders of Eli Lilly and Company.

## Publisher’s Note

All claims expressed in this article are solely those of the authors and do not necessarily represent those of their affiliated organizations, or those of the publisher, the editors and the reviewers. Any product that may be evaluated in this article, or claim that may be made by its manufacturer, is not guaranteed or endorsed by the publisher.
