# Supplementary material for: Abemaciclib in Combination With Endocrine Therapy for Patients With Hormone Receptor-Positive, HER2-Negative Metastatic Breast Cancer: A Phase 1b Study
Source: Front Oncol. 2022 Feb 10;11:810023. doi: 10.3389/fonc.2021.810023 (PMC8868006; doi:10.3389/fonc.2021.810023)

Supplementary Material

# Supplementary Figures

**Supplementary Figure 1.** Mean (standard deviation) plasma concentrations vs. time profiles of letrozole (A), anastrozole (B), tamoxifen (C), and exemestane (D) following administration of abemaciclib with corresponding combination endocrine therapy.


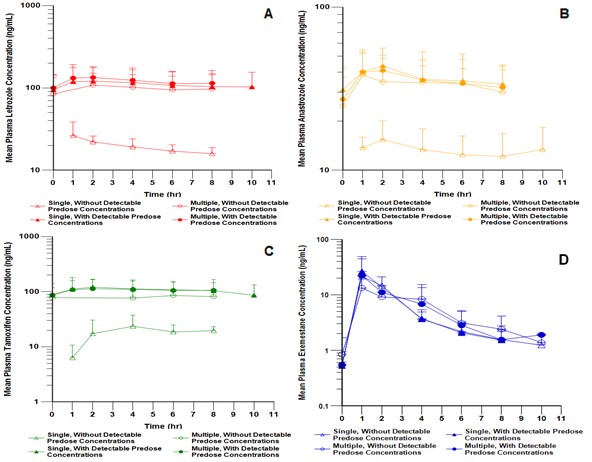


**Supplementary Figure 2.** **Mean MDASI change from baseline, including all post-baseline treatment cycles for each MDASI item. MDASI change scores range from -10 (symptom improvement, less interference) to +10 (symptom worsening, more interference). Parts A, B, C, and D combined (n range per item 62 to 63).**


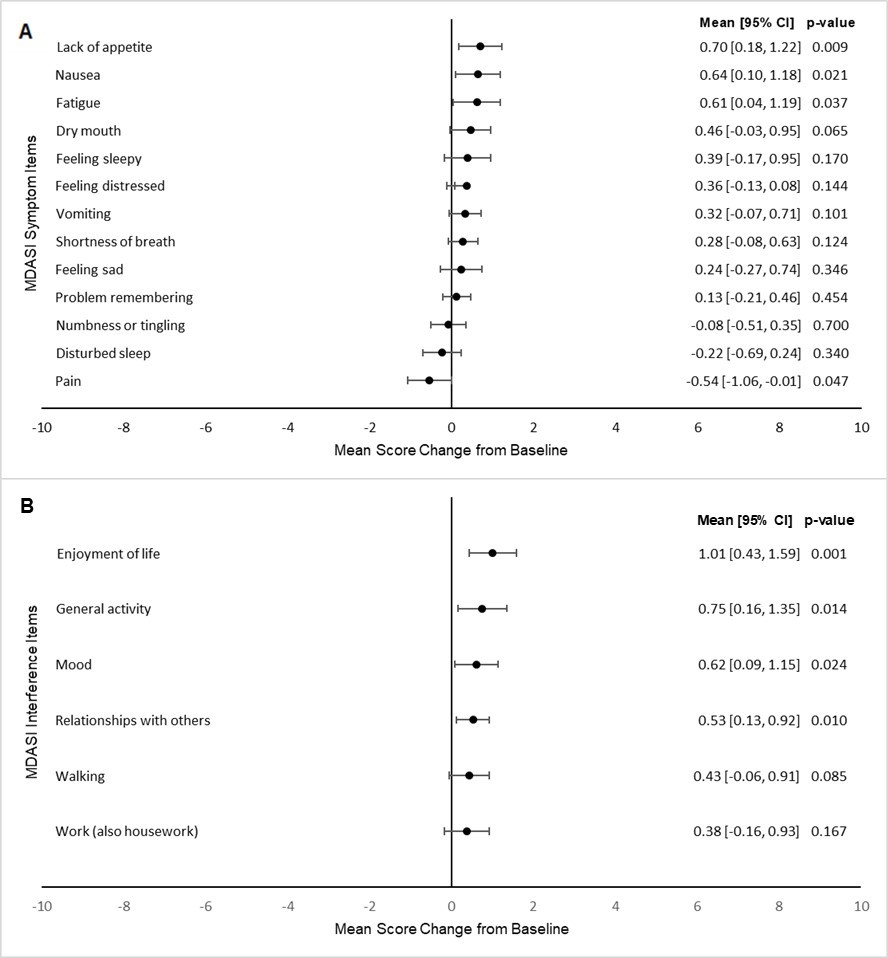

Supplement: Supplementary file 1 [file DataSheet_1.docx]
